# Perceived stress and readiness to undertake biodebridement in the group of nurses undertaking prevention and treatment of chronic wounds

**DOI:** 10.3389/fpubh.2022.1090677

**Published:** 2022-12-22

**Authors:** Joanna Przybek Mita, Dariusz Bazaliński, Rafał Sztembis, Izabela Kuberka, Paweł Więch

**Affiliations:** ^1^Institute of Health Sciences, College of Medical Sciences, University of Rzeszów, Rzeszów, Poland; ^2^Postgraduate Nursing and Midwifery Education Centre, Rzeszów, Poland; ^3^Podkarpackie Specialist Oncology Centre, Specialist Hospital in Brzozów, Brzozów, Poland; ^4^Institute of Medical Sciences, College of Medical Sciences, University of Rzeszów, Rzeszów, Poland; ^5^Faculty of Health Sciences, Wroclaw Medical University, Wroclaw, Poland; ^6^Department of Angiology, University Clinical Hospital, Wroclaw, Poland; ^7^Institute of Health Protection, State University of Applied Sciences in Przemyśl, Przemyśl, Poland

**Keywords:** nurse, chronic wound, debridement, stress, *Lucilia sericata*

## Abstract

**Introduction:**

Stress as the body's response to adverse stimulus is recognized as one of the key problems in basic and clinical neurological studies. Stress is an indispensable component of modern nursing with its low doses being desirable, however, prolonged stress is detrimental to health increasing the risk of chronic disease i.e., hypertension, cardiovascular diseases, electrolyte disturbances, occupational burnout, depression, anxiety disorders. The implementation of certain techniques and therapeutic methods may have a stressful effect from the point of view of practice and the patient's condition.

**Methods:**

The authors undertook the study to assess the intensity of the perceived stress in the group of nurses dealing with wound care in the perspective of implementing wound debridment using *Lucilia sericata* larvae. The study group consisted of 290 nurses specialized in chronic wounds undergoing training at the Postgraduate Training Center of Nurses and Midwives in Rzeszów, out of the entire group of 1.136 individuals participating in training courses organized in Poland in 2020–2021. The study used the diagnostic survey method, the research tool was a scientific research protocol consisting of tools (MDT perception questionnaire, perceived stress scale (PSS-10).

**Results:**

Certain differences in the level of stress in the study sample were observed between age categories, but they were not statistically significant. The greatest differences between the levels of stress in the study sample were observed between people who completed treatment courses and those who did not complete these courses. The higher the stress level, the lower the willingness to undertake such therapy. The analysis of grouped data leads to interesting observations. There were statistically significant differences in the score in the MDT10 scale in the categories of people with different stress levels. The highest readiness to implement MDT was observed in the category of people with the lowest perceived level of stress.

**Conclusions:**

The level of professional experience related to postgraduate education increases opportunities addressing new professional challenges. The level of perceived stress may influence decisions related to the use of biological therapy. The higher the level of stress, the lower the readiness to undertake MDT.

## 1. Introduction

For many years, the issue of stress has been of interest to both psychologists and representatives of medical sciences (1). While this phenomenon is the main concept for understanding both life and evolution, physiologically it is fundamental to survival in the face of disturbed homeostasis, which should trigger adaptive responses. The organism's nonspecific response can be triggered by a wide variety of events. The stimuli that burden the body have been called stressors, and the stress has come to be defined as the non-specific reaction of the body to all demands placed on it. The studies showed that the intensity and duration of stressors determine the stress response and its consequences, it should be kept in mind that experiences in similar situations, innate features and surrounding conditions influence the emergence of stress (2). Currently, the issue of stress is often described and investigated in the context of the classical relational theory of stress, which assumes the importance of the interaction between a person in its multifaceted complexity and their equally complex environment (3). In this context, it is indicated that there is no linear relationship between the stress stimulus and the organism's response. Not only that, the importance of a given stimulus on a person is a derivative of many factors, including resources, thanks to which it is possible not only to cope with stress, but often to reevaluate stressful events into neutral events. Regardless of everything, it is crucial that stress that exceeds the human adaptation abilities, resources, or limits determined by psychobiological predispositions in specific conditions of the broadly understood life environment, may lead to a number of negative consequences, both in the short and long perspective (4).

Strong negative emotions may predispose to disorders of the brain's neurophysiological processes and increase the risk of depression, anxiety and post-traumatic stress disorder (5–7). Stress related to negative emotions provokes defensive reactions, while stress related to positive emotions stimulates the pursuit of goals. The signs of excessive activity include nervousness, haste and problems with concentration, while the signs of in-sufficient activity are mainly fatigue and lack of interest (boredom) (8).

Requirements related to raising the level of knowledge, attitudes and skills are created in the course of graduate education in nursing studies. Psychophysical availability, sensitivity, empathy in ensuring the safety of patients and high-quality medical care result from personality and opportunities created by environmental conditions. The specialist nursing practice has a multi-task character related to taking therapeutic decisions, providing care and therapeutic activities independently in various conditions, often outside the hospital (9). However, it should be borne in mind that functioning in the profession also depends on the ability to adapt to strong stressors, such as experiences related to human suffering, wounds in the injured body, the smell of necrotic tissue, secretions, excretions, chronic and cancerous wounds (10). Nurses in their daily work often struggle with difficult situations. The ability to overcome obstacles and cope with excessive emotional tension in difficult situations may affect the perception of care, the assessment of professional attitude and the success of the therapeutic process. The analysis of the literature on the subject indicates that the stress associated with professional activity is related to the subject of profession, but also work organization and often inappropriate working conditions (11, 12). Stressful work contributes to changes in the mental sphere, as well as fatigue manifested by impairment of general psychomotor activity, deterioration of wellbeing, increased mental tension and sharpened emotional reactions. In addition, disturbances in intra-body reactions, psychosomatic disorders and neurotic reactions may occur. The demands placed on nurses weigh on not only their physical but also mental health. This, in turn, may result in the loss of professional and general life satisfaction (13). It should also be remembered that in a situation of long-term involvement in strenuous work and the emergence of failures in achieving goals, the requirements of the environment may exceed the psychological abilities of a human (14, 15), which in turn may cause a threat to self-efficiency, lower self-esteem and adversely affect on the quality of life (16). This plays a significant role in the development of occupational burnout due to exhaustion from overwork and dissatisfaction with work (17).

Stress is an indispensable component of modern nursing and is desirable in low doses, however, long-term stress is detrimental to health, increasing the risk of chronic disease i.e., hypertension, cardiovascular dis-eases, electrolyte disturbances, burnout, depression, anxiety disorders (1, 2, 12, 16). Occupational stress in the group of nurses negatively determines their quality of life, and thus indirectly affects the quality of services provided, which is associated with a reduction in the quality of care for patients. The intensity and variety of stressors influencing the course of advanced professional practice may determine lower decision-making as well as fears resulting from making therapeutic decisions (12, 18). Nurses, having close contact with patients for whom they are responsible, very often undertake and perform therapeutic activities, independent decisions related to the assessment of health condition, administering medication, and local treatment of wounds. The implementation of certain techniques and therapeutic methods may have a stressful effect from the point of view of practice and the patient's condition. The authors undertook the study to assess the intensity of the perceived stress in the group of nurses dealing with wound care in the perspective of implementing wound debridment using *Lucilia sericata* larvae, because in this case the very nature of the therapy can itself generate a large, additional level of stress.

## 2. Materials and methods

### 2.1. Ethics

The study protocol was approved by the ethics committees of the involved institution (Bioethics Commission at the University of Rzeszow: Resolution no. 2018/01/07 on 07 January 2018). Moreover, the guidelines of the Helsinki Declaration were followed in the course of the conducted research. The participants were informed of the purpose of the study and could withdraw at any time without giving any reason.

### 2.2. Subjects

The study group consisted of 290 nurses specialized in chronic wounds undergoing postgraduate education at the Postgraduate Training Center of Nurses and Midwives in Rzeszów. The study was voluntary. The purpose and structure of the questionnaire were discussed with the designated investigator from the research team. Out of the entire group of 1.136 individuals participating in training courses organized in Poland in 2020–2021 by Postgraduate Training Center for Nurses and Mid-wives branch in Rzeszów, 355 people were enrolled in the study of whom 290 who fully completed questionnaires were subjected to statistical analysis.

### 2.3. Assessments

In the last decade, the interest of researchers and clinicians in *L. sericata* larval excretion/secretion (ES) has increased, and more and more protocols using maggot debridement therapy (MDT) are being implemented in the process of localized wound healing. The use of biosurgery in wound healing processes is becoming more and more frequent in local management recommended by scientific societies (19). Nurses are the most numerous group of medical professionals involved in the prevention and treatment of wounds. The appearance, movement of the larvae and their smell, which is characteristic of the course of therapy, can be perceived un-pleasant, causing reluctance and disgust to use this method in a group of nurses competent to treat wounds. The first experiences of nurses with MDT therapy are described as stressful situations in the form of a multitude of thoughts, there were problems with falling asleep mainly related to the images of worms wiggling in the wound. It was assumed that the level of perceived stress and the deficit of knowledge about the method may reduce the potential readiness to implement MDT in therapeutic protocols.

### 2.4. Statistical analysis

The data collected in this study were analyzed with IBM SPSS Statistics 21 for Windows. The statistical significance level was set at *p* = 0.05. The reliability of the scales were tested with the Cronbach alpha. In order to evaluate the variables distributions descriptive statistics were applied. Normality of the distribution was tested with Kolmogorov–Smirnov test. The Pearson chi square test was performed to test differences between classes of variables. The Spearman rho rank correlation was used to assess the correlation between quantitative variables.

### 2.5. Research tools

The study used the diagnostic survey method, the research tool was a research protocol consisting of three parts (questionnaires). The first part concerned the sociodemographic data of the respondents, seniority and postgraduate qualifications entitling to wound treatment. The second part contained abbreviated versions of the MDT 10 perception questionnaire designed to assess the readiness to implement MDT (Maggot Debridement Therapy) in the treatment of chronic wounds, developed by D. Bazaliński, A. Krawiec: consisting of 10 items divided into two subscales (knowledge, motivation). The responses to questions constructed on a five-point Likert scale (I strongly disagree, I disagree, I have no opinion, I agree, I strongly agree). For each positive answer, the respondent received 5 points, min. 10 points, max. 50 points, the higher the number of points, the greater the level of readiness to implement MDT. The reliability of the MDT 10 perception assessment questionnaire was determined at the level of Cronbach's Alpha level α = 0.76 (20). The third tool used in the course of the study was the perceived stress scale (PSS-10) by Cohen et al. in Polish adaptation by Juczyński and Ogińska-Bulik. PSS-10 containing 10 items on various subjective feelings related to personal problems and events, behaviors and coping methods, is used to assess the intensity of stress related to one's own life situation over the last month. It contains 10 questions on various subjective feelings related to problems and personal events, behaviors and ways of coping. The results in the questionnaire range from 0 to 40 points. The higher the score, the higher the level of perceived stress. The tool allows to determine the level of perceived stress a month prior to the examination (21, 22).

### 2.6. Characteristics of the respondents

The study included 290 nurses practicing in Poland authorized to treat wounds and perform therapeutic procedures in this respect. The majority of the study sample were women (87.9%). The mean age was over 40, the median was 44. Median of work experience amounted to 18 years. Most (79.0%) of the respondents declared higher education (MSc or BSc in Nursing), followed by the graduates of High School of Nursing (21.0%) (Table 1).

**Table 1:** Demographic characteristics of the respondents.

		***N***	**%**
Sex	**Total**	**290**	**100.0%**
	Female	255	87.9%
	Male	35	12.1%
Age	24–34	77	26.6%
	35–44	74	25.5%
	45–54	105	36.2%
	55–64	34	11.7%
Education^*^	Registered nurse	61	21.0%
	BSc in nursing	71	24.5%
	MSc in nursing	158	54.5%
Work experience in the profession of a nurse	1–5 years	52	17.9%
	11–15 years	33	11.4%
	16–20 years	59	20.3%
	21–30 years	61	21.0%
	More than 30 years	54	18.6%

* Nursing education in Poland is diversified. Since the accession to the European Union in 2004 education is provided at the level of first and second degree studies. Previously it was provided in post-secondary schools and vocational high schools.

## 3. Results

### 3.1. The level of perceived stress in the studied group of nurses

**Figure 1:**
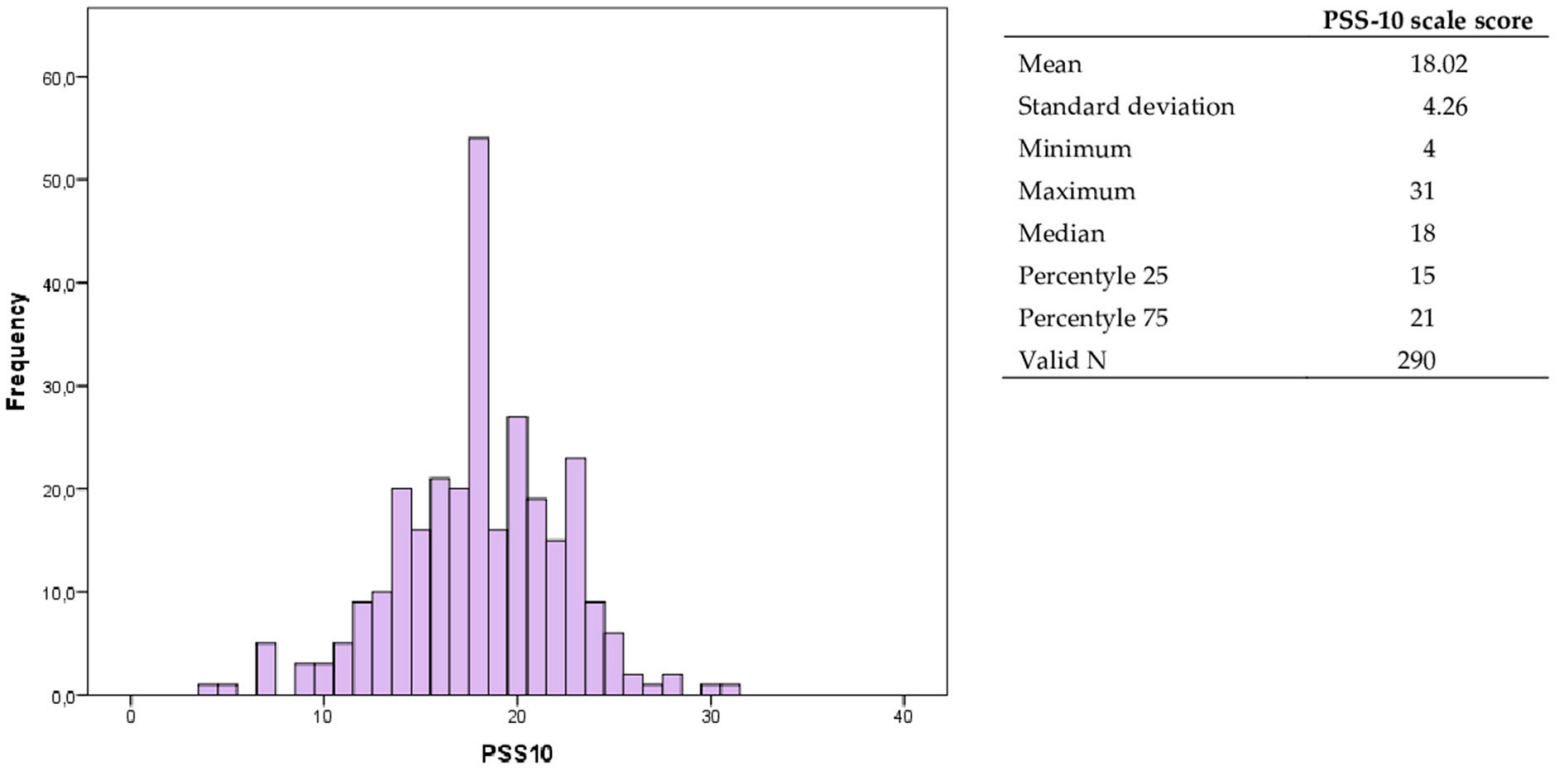
Histogram of the PSS-10 scale.

The level of perceived stress was assessed on the basis of the PSS-10 scale. The obtained results in the group of nurses and practitioners dealing with prophylaxis and treatment of wounds indicate average and high values of the perceived stress (*df* = 290, *p* < 0.001) (Figure 1). The continuous scale was converted into a sten scale and grouped into 3 categories: low, average and high stress levels. The conversion was performed on the basis of the norms proposed by Z. Juczyński. A low level of perceived stress was noted in 37 subjects (12.8%), average in 147 (50.7%), high in 106 (36.6%).

### 3.2. Selected variables influencing stress

The data on selected sociodemographic variables (age, gender, and education) and the stress level according to PSS-10 were compiled. The highest values of the stress level were observed in the group of the youngest respondents (24–34 years old) and the oldest (55–64 years old). In turn, the largest share of people with the lowest level of stress was recorded in the intermediate age categories (35–44 and 45–54 years) (Chi-square = 13,334, *df* = 6, *p* < 0.05) (Table 2). In the gender category, slightly higher values of the stress level were observed among men than among women, but the differences were not statistically significant (Chi-square = 1.015, *df* = 2, *p* > 0.05.).

**Table 2:** Stress level with respect to age.

	**Age**				**Total**
	**24–34**	**35–44**	**45–54**	**55–64**	
Low stress levels	10.4%	17.6%	15.2%	0.0%	12.8%
Average stress levels	44.2%	50.0%	56.2%	50.0%	50.7%
High stress levels	45.5%	32.4%	28.6%	50.0%	36.6%
Total	77	74	105	34	290

Chi-square = 13.334, df = 6, *p* < 0.05.

The work experience of the respondents ranged from 1 to 42 years. The median was 18 years. The distribution of seniority differed significantly from the normal distribution. In order to check the relationship between the level of perceived stress and the length of service in the nursing profession, the Spearman's rho correlation coefficient between these variables was calculated. The obtained coefficient was not statistically significant, which indicated no relationship between work experience and the level of perceived stress. In order to illustrate the distribution of the stress level in relation to the length of service, grouped variables were presented in Table 3. Certain differences in the level of stress in the study sample were observed between age categories, but they were not statistically significant.

**Table 3:** Stress level and work experience in the nursing profession.

**Stress level**		**Work experience in the nursing profession**				**Total**
		**1–9 years**	**10–19 years**	**20–29 years**	**30**+ **years**	
Low stress levels	*N*	10	8	13	6	37
	%	14.3%	9.5%	17.8%	9.5%	12.8%
Average stress levels	*N*	29	45	33	40	147
	%	41.4%	53.6%	45.2%	63.5%	50.7%
High stress levels	*N*	31	31	27	17	106
	%	44.3%	36.9%	37.0%	27.0%	36.6%
Total	*N*	70	84	73	63	290
	%	100.0%	100.0%	100.0%	100.0%	100.0%

Chi-square = 9.315, df = 6, *p* > 0.05.

Data on the type of education and the level of perceived stress were compared. For the purposes of the study, a division was made according to the type of education (due to changes in the nursing education system, the level of education is related to the age of the respondents). In order to investigate the relationship between stress and education, the analysis was performed only in the older group of nurses, i.e., in the group where there is no significant correlation between age and education (in the group of people aged 45–64). Despite certain differences in the level of stress in terms of education in the study group, no statistically significant relationships were found (*p* > 0.05). The hypothesis that the education of the respondents could determine the level of perceived stress was rejected (Table 4).

**Table 4:** Completed education and the level of perceived stress.

**Stress level**		**Education**			**Total**
		**Medical vocational high school/post-secondary school (certified nurse)**	**BSc in nursing**	**MSc in nursing**	
Low stress levels	*N*	5	1	9	15
	%	10.9%	2.9%	15.8%	10.9%
Average stress levels	*N*	30	19	26	75
	%	65.2%	55.9%	45.6%	54.7%
High stress levels	*N*	11	14	22	47
	%	23.9%	41.2%	38.6%	34.3%
Total	*N*	46	34	57	137
	%	100.0%	100.0%	100.0%	100.0%

Chi-square = 7.231 [11.1% cells (1) has the expected number below 5], df = 4, *p* > 0.05.

It was checked whether the most frequently taken courses and specializations had an impact on the level of perceived stress. Table 5 summarizes the values of the stress level depending on the completion or absence of wound treatment courses, qualification courses and specialization. The greatest differences between the level of stress in the study sample were observed between people who completed treatment courses and those who did not complete these courses. Those who did not complete wound healing courses were slightly more likely to have high levels of stress than those who did not complete such courses. However, these differences, as in the case of qualification and specialization courses, were not statistically significant (*p* > 0.05). Therefore, based on the available data, it cannot be concluded that the analyzed courses and specializations have an impact on the level of stress. It has been observed that high levels of stress were more common in the respondents who declared completing one or two types of courses and specializations than in the categories of people who declared three or more types. Therefore, it can be concluded that systematic education and improvement of qualifications positively determined the level of stress. Differences in the level of stress between the categories of people with different number of courses and specializations completed were statistically significant (Chi-square = 17.722, *df* = 6, *p* < 0.05.).

**Table 5:** Completed postgraduate training and the level of perceived stress.

**Stress level**	**Types of courses and specialization completed**						**Total**
	**Wound treatment**		**Qualification course**		**Specialization**		
	**No**	**Yes**	**No**	**Yes**	**No**	**Yes**	
Low stress levels	17	20	18	19	15	22	37
	15.9%	10.9%	12.6%	12.9%	11.0%	14.4%	12.8%
Average stress levels	45	102	70	77	71	75	146
	42.1%	55.7%	49.0%	52.4%	52.2%	49.0%	50.5%
High stress levels	45	61	55	51	50	56	106
	42.1%	33.3%	38.5%	34.7%	36.8%	36.6%	36.7%
Total	107	183	143	147	136	153	289
	100.0%	100.0%	100.0%	100.0%	100.0%	100.0%	100.0%
	Chi-square = 5.200, *df* = 2, *p* > 0.05		Chi-square = 0.456, *df* = 2, p > 0.05		Chi-square = 0.776, *df* = 2, *p* > 0.05		

### 3.3. Perceived stress and readiness to undertake biodebridement (MDT)

Readiness to undertake biodebridement was assessed using the direct question (Please specify your readiness for treating of wounds using biological therapy independently) and the MDT 10 questionnaire. There was no correlation *p* > 0.05 between the value on the stress scale (PSS-10) and the MDT10 values. On the other hand, the correlation between PSS-10 and the self-assessment of treating of wounds using biological therapy independently was statistically significant (*r* = −0.224, *p* = 0.037). The level of stress may determine the readiness to undertake activities with the use of biological therapy (Table 6). The higher the stress level, the lower the willingness to undertake such therapy. No statistically significant correlation in the case of the MDT scale measured with the Spearman's rho coefficient meant no monotonic dependence. However, the analysis of grouped data leads to interesting observations. There were statistically significant differences in the score in the MDT10 scale in the categories of people with different stress levels. The highest readiness to implement MDT was observed in the category of people with the lowest perceived level of stress.

**Table 6:** Readiness to implement MDT therapy (MDT 10 scale) and the level of stress intensity.

		**Low stress levels**	**Average stress levels**	**High stress levels**	**Total**	**Pearson Chi-square**
	Total	37	147	106	290	
The use of Maggot Debridement Therapy (MDT) accelerates the debridement of necrotic tissue in the treatment of chronic wounds compared to autolytic and mechanical methods	I disagree, I have no opinion	5.4%	12.2%	10.4%	10.7%	0.082
	I rather agree	27.0%	46.3%	40.6%	41.7%	
	I strongly agree	67.6%	41.5%	49.1%	47.6%	
A single maggot can remove 25 mg of necrotic material from a wound in 24 hours	I disagree, I have no opinion	48.6%	61.2%	34.0%	49.7%	0.001
	I rather agree	32.4%	23.8%	46.2%	33.1%	
	I strongly agree	18.9%	15.0%	19.8%	17.2%	
Brown exudate with a specific smell during MDT therapy is a positive symptom suggesting liquefaction of necrotic tissue by the larvae	I disagree, I have no opinion	29.7%	63.3%	37.7%	49.7%	0.000
	I rather agree	48.6%	27.2%	48.1%	37.6%	
	I strongly agree	21.6%	9.5%	14.2%	12.8%	
5-10 larvae are usually used per 1cm^2^ for wound debridement	I disagree, I have no opinion	43.2%	66.0%	50.0%	57.2%	0.020
	I rather agree	27.0%	18.4%	31.1%	24.1%	
	I strongly agree	29.7%	15.6%	18.9%	18.6%	
Wound edge protection is essential to protect the skin from migration and irritation by larvae defensins (secretions)	I disagree, I have no opinion	13.5%	10.9%	27.4%	17.2%	0.000
	I rather agree	37.8%	61.9%	38.7%	50.3%	
	I strongly agree	48.6%	27.2%	34.0%	32.4%	
I am committed to improving the patient's quality of life and healing the wound that I am dealing with	I disagree, I have no opinion	5.4%	5.4%	8.5%	6.6%	0.015
	I rather agree	10.8%	38.1%	27.4%	30.7%	
	I strongly agree	83.8%	56.5%	64.2%	62.8%	
I am motivated to conduct educational activities so that the patient tolerates MDT as good as possible	I disagree, I have no opinion	10.8%	7.5%	11.3%	9.3%	0.032
	I rather agree	18.9%	46.9%	38.7%	40.3%	
	I strongly agree	70.3%	45.6%	50.0%	50,0.3%	
I change the top dressings and control the wound in such a way to minimize patient's visual contact with the larvae in the wound	I disagree, I have no opinion	24.3%	10.2%	7.5%	11.0%	0.000
	I rather agree	16.2%	55.8%	43.4%	46.2%	
	I strongly agree	59.5%	34.0%	49.1%	42.8%	
I point out the benefits of topical wound therapy with MDT to the patient	I disagree, I have no opinion	10.8%	6.8%	9.4%	8.3%	0.036
	I rather agree	16.2%	44.2%	35.8%	37.6%	
	I strongly agree	73.0%	49.0%	54.7%	54.1%	
I implement MDT in case patient accepts it and clinical indications	I disagree, I have no opinion	21.6%	43.5%	17.0%	31.0%	0.000
	I rather agree	13.5%	21.8%	33.0%	24.8%	
	I strongly agree	64.9%	34.7%	50.0%	44.1%	

## 4. Discussion

Stress related to the performance of health services and therapeutic procedures is an interactive event between the work situation and the person and their coping resources. This situation changes the mental and physiological state of an individual and conditions its functioning. Psychological preparation of representatives of medical professions, who are directly responsible for human life, to function effectively in difficult situations is of particular importance. It allows to conduct effective, high-quality services despite unfavorable circumstances, while on the other hand, despite exposure to long-term stress, protects against the risk of burnout, ensuring a better quality of life (23, 24). Nursing is by definition a stressful profession because it is associated with complex professional requirements and needs and high social expectations (11, 12, 25).

An attempt was made to assess the impact of the intensity of perceived stress on the readiness to undertake MDT therapy by qualified nurses to perform therapeutic procedures in patients with chronic wounds. The undertaken research topic is fascinating since both the shortages in knowledge and skills regarding the use of *L. Sericata* larvae, as well as the lack of experience may predispose to not recommending MDT. On the other hand, it is necessary to take into account the reluctance resulting from the negative perception related to the view and the specific smell that are characteristic of the larvae. Maggot-induced loathing and disgust often stem from associations, negative experiences, and childhood images of decaying carcasses, animal excrement, or rotten garbage. It is associated with unpleasant images and smells, which may cause negative psychosomatic symptoms (20, 26). Our study focused on a group of nurses qualified and experienced in undertaking therapeutic interventions in patients with chronic wounds. In our study (20) concerning readiness to implement biodebridment with the use of *L. Sericata* larvae, the subjects showed motivation but declared a deficit of knowledge, skills and experience in wound treatment with MDT. These variables may result in reluctance as well as some concerns about MDT implementation. Morozow and Sherman (27) drew attention to the perception of patients who declared that the images of maggots were more repulsive than the images of gangrenous wounds. This observation is significant as it indicates that much education and support needs to be put in place to address the concerns and worries of eligible patients.

It was assumed in the conducted study that female gender and the level of perceived stress may be a strong predictor of the reluctance to implement MDT in the protocols of debridement and revitalization of chronic wounds. The obtained results allow for the conclusion that in the gender category, slightly higher values of the stress level were observed among men than among women, however, the differences were not statistically significant. The highest values of the stress level were observed in the group of the youngest respondents (24–34 years old) and the oldest (55–64 years old). On the other hand, the highest share of people with the lowest level of stress was recorded in the intermediate age categories (35–44 and 45–54). The respondents aged 35–54 were qualified and experienced medical staff, most of the representatives of this group declared continuous education and development in the field they represent. The obtained data can be used to draw conclusions based on the hypothesis that professional experience and acquired knowledge reduce the level of perceived stress, resulting from the implementation of new therapeutic procedures. The high level of stress in the youngest and oldest groups is obvious in the context of the stage of working life; the beginning of work, career, introduction to the system, the stage in which the employee is expected to be more effective and confident in the performance of duties, and there may be a fear of meeting these expectations. In the oldest group, the stage of exhaustion with work, loss of motivation to perform work or taking up job or new challenges may prevail.

The authors indicate that advanced nursing practice can improve and strengthen the health care system by maintaining a high standard of services, thereby reducing the final costs associated with treatment time and potential complications. Nurses are responsible for taking preventive measures and local wound treatment strategies in patient care, especially at home. The condition, however, is the continuous updating of knowledge, undertaking reflections in practice, allowing for the assessment of compliance with the current knowledge, recommendations, and the practical use of professional autonomy (28, 29).

Our study indicated that the highest readiness to use MDT was observed in the category of people with the lowest level of stress. However, relatively high values of the MDT10 scale were also noted in the high-stress category. In the context of the complexity of stress phenomenon, its consequences and conditions, this observed dependence may be conditioned by personality factors, which differentiate the studied group in this way. Such a feature is e.g., perfectionism, which in the context of performing medical professions strongly correlates with the level of stress and the risk of occupational burnout (30). Thus, in practice, especially the second group in this specific occupational situation would be even more exposed to stress or burnout, and as a group they would constitute the target group of preventive interventions. The issue of organizing postgraduate training and undertaking activities aimed at equipping nurses with such resources that will allow them to get to know themselves and develop the ability to deal with stress in a more task-oriented manner. The analysis of the obtained data in the context of readiness to undertake biodebridement indicates that the resource understood as education (preparation within the framework of courses and training) is an insufficient variable influencing the level of experienced stress. However, the observed tendency may indicate that building resources to cope with the situation at this level may have a beneficial effect.

Perhaps it is worth conducting research on what elements of education could, in the opinion of practitioners, positively affect the level of perceived stress related to the perception of biodebridement. It is very likely that it is the practical experience of conducting such therapy, giving one a greater sense of control over own work, that can significantly reduce the level of perceived stress. Postgraduate education is not only about providing knowledge and practice that will allow the professionals to approach the performance of various duties in a task-oriented manner. Acquiring knowledge and acquiring new practical skills should be carried out under the supervision of a mentor, a person with knowledge and practical experience. This, of course, will not eliminate stress completely, but with the inclusion of elements supporting and motivating getting to know oneself into postgraduate education, including personal and other conditions for coping with daily work tasks, it can potentially reduce the level of stress and positively affect the quality of services provided and quality of life.

Development of nursing occurs through leaders who do mentoring to younger practitioners. Mentoring itself is perceived as a certain human development strategy based on a relationship between a person with extensive experience in a certain field and a person who wants to gain experience. This issue is based on leadership and inspiration based on a dialogue that formulates specific goals for the student to motivate. High level of commitment, job satisfaction, lower percentage of burnout, professional prestige, and professional development are the most frequently described benefits of the mentoring system (31, 32). High therapeutic benefits and practical implementation of the therapy by nursing students in the treatment of wounds show that mentoring contributes to the development of clinical nursing (33, 34), and therefore should be used in the situation of training and implementation of MDT in clinical practice.

The results of the study carried out by our team indicate that the implementation of new, little-known procedures in nursing can be burdened with a lot of stress and fears related to making a mistake. The ability to motivate should be perceived as one of the most important managerial functions, which requires the use of comprehensive and integrated systems to undertake and implement the expected goals, functions and tasks. Discussing cases and problem solving, supporting staff in performing activities for the benefit of the patient, as well as raising awareness of the need for own professional development are directions of potential future research. In our study, we assessed the readiness to implement MDT, the obtained results should be a premise for motivating staff performing advanced nursing practices. Incentive programs and evaluation should be considered as the next stage of research related to the implementation of MDT in clinical practice.

Worldwide, MDT therapy is used to a limited extend, although many studies confirm its benefits and efficacy in the treatment of chronic wounds. As chronic wounds are becoming more common, and MDT may play a key role in the local healing of wounds. This incomplete utilization may be related to the perception of staff as well as of the patients themselves. Healthcare professionals and especially nurses are key in implementing and encouraging patients to accept the MDT. Promoting and showing the positive benefits of the method and educating patients can be beneficial in resolving treatment misconceptions.

### 4.1. Limitations

The presented study focuses on selected variables (age, education, work experience, gender) having a potential impact on the level of perceived stress and its impact on the readiness to implement MDT practice, no verification of variables related to family life and burdens and perception of professional work was performed. Another limitation of the study is the lack of consideration of cultural components. It should be assumed that there may be differences in coping with stress and tolerance of the sight of larvae used in the wound treatment process between Eastern and Western cultures.

## 5. Conclusions

The level of professional experience related to postgraduate education increases the possibility of taking up new professional challenges. The level of perceived stress may influence decisions related to the use of biological therapy. The higher the stress level, the lower one's willingness to undertake MDT.

## Data availability statement

The original contributions presented in the study are included in the article/supplementary material, further inquiries can be directed to the corresponding author.

## Author contributions

Conceptualization and data curation: JP and DB. Methodology: PW, IK, and JP. Formal analysis: RS, PW, and DB. Investigation: DB and PW. Writing—original draft preparation: JP, DB, and IK. Writing—review and editing: JP, DB, and PW. Supervision: DB, JP, RS, and PW. All authors have read and agreed to the published version of the manuscript.
